# Bilateral Anophthalmia Associated With Ventricular Septal Defect, Atrial Septal Defect, and Patent Ductus Arteriosus: A Rare Neonatal Case Report

**DOI:** 10.1155/crpe/4616554

**Published:** 2026-09-27

**Authors:** Abdullahi Ali Roble, Abdisalam Ismail Hassan, Said Abdi Mohamed, Hassan Muhumed Mohamed, Ishak Ahmed Abdi, Abdijalil Abdullahi Ali, Balqiz Mohamed Kulle, Ahmed Shafie Aden, Hanan Hassan Hirei Abdalla, Mohamed Osman Dahir Alasow, Shuayb Abdullahi Adan, Ahmed Muhamad Bashir

**Affiliations:** ^1^ Ophthalmology Department, Mogadishu Somali–Türkiye Recep Tayyip Erdoğan Training and Research Hospital, Mogadishu, Somalia; ^2^ Faculty of Medicine and Health Sciences, Jamhuriya University of Science and Technology, Mogadishu, Somalia, just.edu.so; ^3^ Neurology Department, Mogadishu Somali–Türkiye Recep Tayyip Erdoğan Training and Research Hospital, Mogadishu, Somalia; ^4^ Cardiology Department, Mogadishu Somali–Türkiye Recep Tayyip Erdoğan Training and Research Hospital, Mogadishu, Somalia; ^5^ Cardiovascular Surgery Department, Mogadishu Somali–Türkiye Recep Tayyip Erdoğan Training and Research Hospital, Mogadishu, Somalia; ^6^ Anesthesia and Reanimation Department, Mogadishu Somali–Türkiye Recep Tayyip Erdoğan Training and Research Hospital, Mogadishu, Somalia; ^7^ Radiology Department, Mogadishu Somali–Türkiye Recep Tayyip Erdoğan Training and Research Hospital, Mogadishu, Somalia; ^8^ Internal Medicine Department, Somali Sudanese Specialized Hospital, Mogadishu, Somalia; ^9^ Research and Education Unit, Mogadishu Virtual Academy (MOVA), Mogadishu, Somalia

**Keywords:** atrial septal defect, bilateral anophthalmia, congenital heart disease, neonate, patent ductus arteriosus, resource-limited setting, ventricular septal defect

## Abstract

**Background:**

Anophthalmia is a rare congenital ocular malformation characterized by the complete absence of the eye and its associated ocular structures. Bilateral anophthalmia is particularly uncommon and is frequently associated with multisystem congenital anomalies. Congenital heart disease represents one of the most commonly reported systemic associations; therefore, all infants diagnosed with bilateral anophthalmia should undergo comprehensive systemic evaluation, including detailed cardiovascular assessment. However, the coexistence of bilateral anophthalmia with multiple congenital cardiac defects remains uncommon, with only a limited number of cases reported in the literature.

**Case Presentation:**

We report the case of a 3‐day‐old full‐term Somali male neonate, born in a resource‐limited rural setting, who presented with the complete bilateral absence of the ocular globes, which was identified immediately after birth. Ophthalmologic examination confirmed bilateral congenital anophthalmia. During hospitalization, the infant developed clinical features of hemodynamically significant left‐to‐right shunt physiology, including tachypnea during feeding, feeding difficulties, easy fatigability, and diaphoresis. Brain magnetic resonance imaging confirmed complete bilateral anophthalmia without associated intracranial abnormalities. Transthoracic echocardiography demonstrated multiple congenital cardiac defects, including a 4‐mm muscular ventricular septal defect, a 4‐mm secundum atrial septal defect associated with an atrial septal aneurysm, and a 3‐mm patent ductus arteriosus with restrictive left‐to‐right shunting. Owing to the hemodynamic significance of these lesions and the lack of advanced pediatric cardiac surgical services locally, the patient was stabilized and referred abroad for definitive surgical management.

**Conclusion:**

This case underscores the rarity of bilateral congenital anophthalmia associated with multiple congenital cardiac anomalies and highlights the critical need for thorough systemic evaluation of neonates with major congenital malformations. Early multidisciplinary involvement and timely referral to specialized centers are essential, particularly in resource‐limited settings where diagnostic and therapeutic options are constrained.

## 1. Introduction

Anophthalmia is a rare congenital ocular malformation characterized by the complete absence of the eye and associated ocular structures within the orbit, with an estimated prevalence of 1 in 10,000–20,000 live births, depending on regional and methodological variations [1]. It results from failure of normal ocular development or regression of the developing optic vesicle [2]. The condition was first described by Lycosthenes in 1547 and later by Schenk in 1609 [3]. Mann subsequently classified anophthalmos into three types: primary, secondary, and consecutive (degenerative) anophthalmos [4].

Primary anophthalmia results from failure of optic vesicle formation due to arrested ocular development during the early fourth week of gestation and occurs in approximately 0.18–0.4 per 10,000 live births [5]. It is characterized by the absence of the globe and may be accompanied by the absence or severe underdevelopment of associated ocular and optic pathway structures. Secondary orbital abnormalities may also occur, including reduced orbital growth, abnormalities of the extraocular muscles, and narrowing of the optic foramen as a consequence of disrupted intrauterine development [5]. In contrast, degenerative anophthalmia occurs when the eye initially develops but subsequently regresses, potentially because of vascular disruption or other developmental insults affecting the ocular and periocular tissues [5].

Diagnosis typically begins with clinical recognition of absent ocular structures in the neonatal period and is complemented by imaging to confirm the diagnosis and identify associated abnormalities. Magnetic resonance imaging (MRI) is the preferred imaging modality because it provides detailed visualization of the orbital soft tissues, optic pathways, and intracranial structures without exposing the infant to ionizing radiation. Computed tomography (CT) should generally be reserved for selected cases requiring detailed evaluation of the bony orbit or when MRI is unavailable. Given the strong genetic contribution to anophthalmia, genomic investigations, including chromosomal microarray analysis and whole‐exome sequencing, may help identify underlying chromosomal abnormalities, pathogenic variants, and syndromic associations, thereby supporting diagnostic evaluation and genetic counseling [6].

Bilateral anophthalmia may occur in isolation or in association with congenital abnormalities involving multiple organ systems, including the cardiovascular system. Congenital heart disease (CHD) is the most common congenital anomaly worldwide, affecting approximately 8 per 1000 live births. Ventricular septal defects (VSDs), atrial septal defects (ASDs), and patent ductus arteriosus (PDA) are among the commonly encountered congenital cardiac lesions and may contribute to clinically significant left‐to‐right shunt physiology [7].

The coexistence of bilateral anophthalmia with multiple congenital cardiac abnormalities is rarely reported and raises the possibility of shared developmental or genetic mechanisms. Such an association also underscores the importance of comprehensive systemic evaluation in neonates presenting with major congenital ocular malformations. In resource‐limited settings, inadequate antenatal screening, incomplete perinatal documentation, and limited access to advanced diagnostic and specialized surgical services may further complicate diagnosis and management. We report a neonate with bilateral congenital anophthalmia associated with a muscular VSD, a secundum ASD with an atrial septal aneurysm, and a PDA, highlighting the importance of comprehensive systemic evaluation, multidisciplinary management, and timely referral to specialized care in a resource‐limited setting.

## 2. Case Presentation

A 3‐day‐old full‐term Somali male neonate, born in a village near Mogadishu, was referred to our tertiary care hospital after the absence of both eyes was noted immediately after birth. He had been delivered by spontaneous vaginal delivery to nonconsanguineous parents. Apgar scores and documentation of the immediate postnatal assessment were unavailable. The pregnancy was reportedly uneventful, with no history of maternal illness, medication use, radiation exposure, or TORCH infections. The neonate was the youngest of four siblings, all of whom were alive and had no known congenital anomalies. The mother was 40 years old at the time of delivery, representing advanced maternal age. There was no known family history of congenital malformations or genetic disorders.

Upon arrival, the neonate was admitted through the pediatric emergency department for further evaluation and stabilization. Initial assessment showed a hemodynamically stable infant with normal body temperature and oxygen saturation. Ophthalmologic examination revealed bilaterally hypoplastic, diminutive eyelids with the complete absence of visible ocular globes (Figure 1). Direct ophthalmoscopy did not identify any ocular tissue, consistent with bilateral congenital anophthalmia.

**FIGURE 1:**
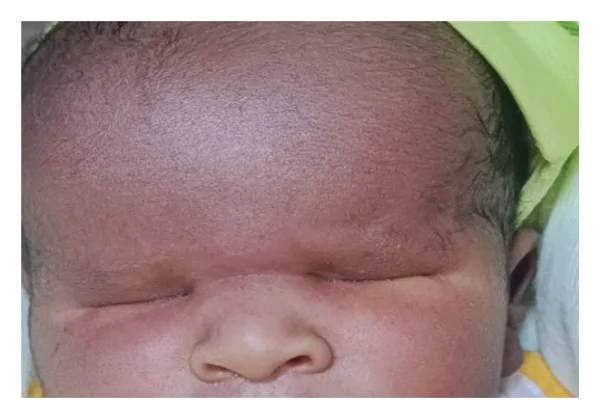
Clinical photograph of a newborn demonstrating bilateral anophthalmia, characterized by the absence of visible ocular structures.

During hospitalization, the infant developed clinical features suggestive of significant left‐to‐right cardiac shunt physiology, including tachypnea during feeding, feeding difficulties, easy fatigability, and excessive sweating while breastfeeding. No central or peripheral cyanosis was observed. Cardiovascular examination revealed a hyperdynamic precordium, a pansystolic murmur best heard at the left lower sternal border, and a continuous murmur over the left infraclavicular region. Respiratory examination demonstrated mild tachypnea without chest retractions or other signs of respiratory distress. Abdominal and neurological examinations were unremarkable. A comprehensive dysmorphologic examination revealed no craniofacial dysmorphic features apart from the ocular abnormalities. The external ears were normally formed and appropriately positioned, with no limb, digital, genital, or other apparent external congenital abnormalities identified.

Laboratory investigations were performed to evaluate for congenital infections. Serologic testing was negative for toxoplasma IgM and IgG, rubella IgG, cytomegalovirus (CMV), herpes simplex virus (HSV), and syphilis, with no laboratory evidence of congenital infection. Renal function tests showed a blood urea level of 77 mg/dL and a serum creatinine level of 0.88 mg/dL. Brain MRI confirmed bilateral anophthalmia, demonstrating complete absence of both ocular globes without associated intracranial abnormalities (Figure 2). Additional sagittal and coronal MRI images further demonstrated the absence of the ocular globes within their expected anatomical locations (Figure 3A,B). Abdominal ultrasonography revealed no abnormalities.

**FIGURE 2:**
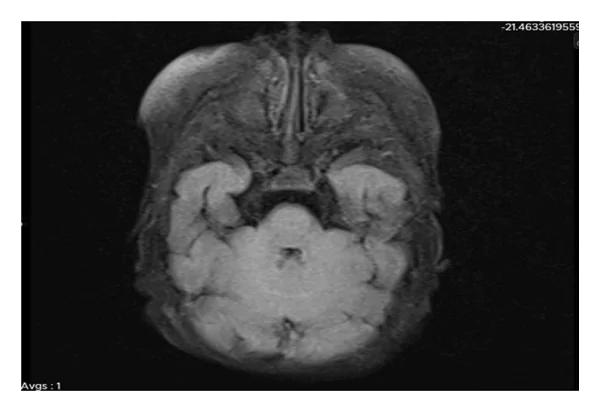
Axial MRI image demonstrating the absence of the bilateral ocular globes (arrows), consistent with bilateral anophthalmia.

**FIGURE 3:**
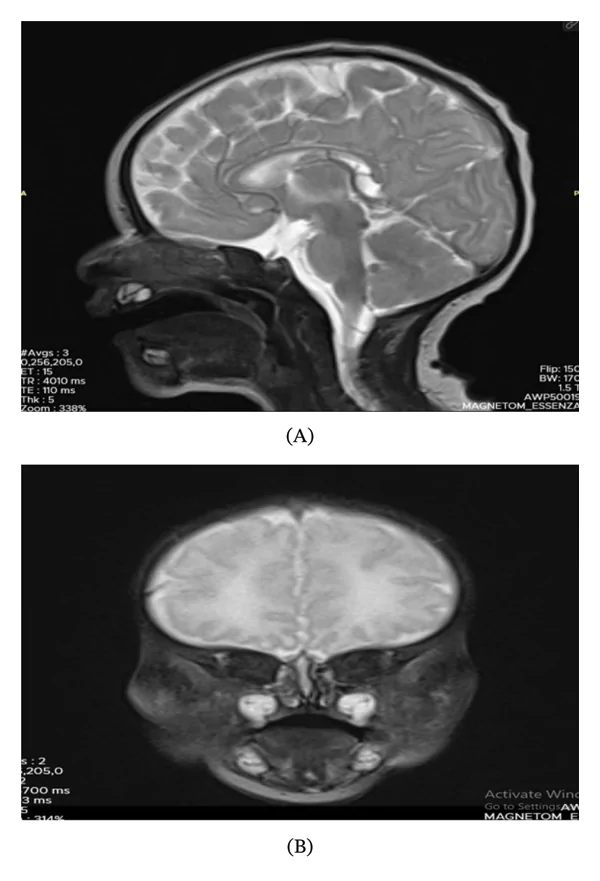
(A) Sagittal MRI image demonstrating the absence of the ocular globes in their normal anatomical positions. (B) Coronal MRI image demonstrating the absence of the ocular globes within the orbital cavities.

Given the cardiopulmonary symptoms and abnormal cardiovascular examination findings, the patient underwent comprehensive transthoracic echocardiography. This demonstrated multiple congenital cardiac abnormalities, including a 3‐mm PDA with restrictive left‐to‐right shunting into the pulmonary artery (Figure 4), a 4‐mm secundum ASD associated with an atrial septal aneurysm (Figure 5), and a 4‐mm muscular VSD (Figure 6). Collectively, these cardiac defects were considered clinically significant in the context of the infant’s symptoms of tachypnea during feeding, feeding difficulties, easy fatigability, and diaphoresis.

**FIGURE 4:**
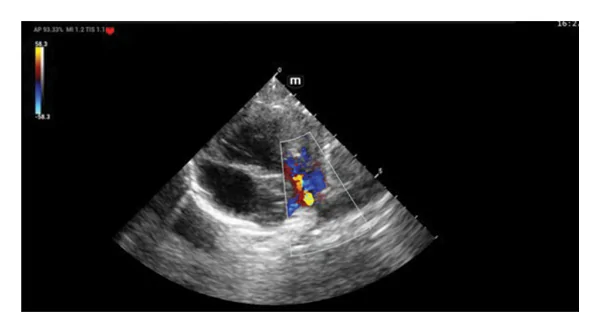
Parasternal short‐axis echocardiographic view demonstrating a 3‐mm patent ductus arteriosus (PDA) with restrictive left‐to‐right flow into the pulmonary artery. The blue arrow indicates the PDA.

**FIGURE 5:**
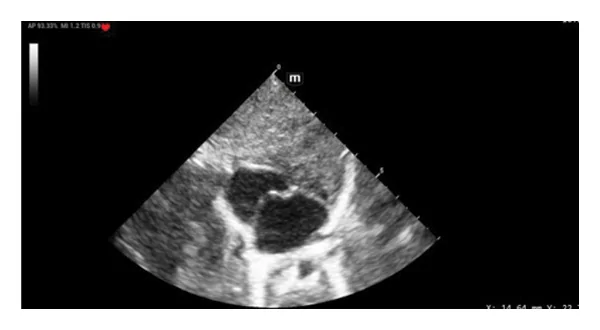
Subxiphoid echocardiographic view demonstrating a 4‐mm secundum atrial septal defect (ASD) associated with an atrial septal aneurysm. The blue arrow indicates the ASD.

**FIGURE 6:**
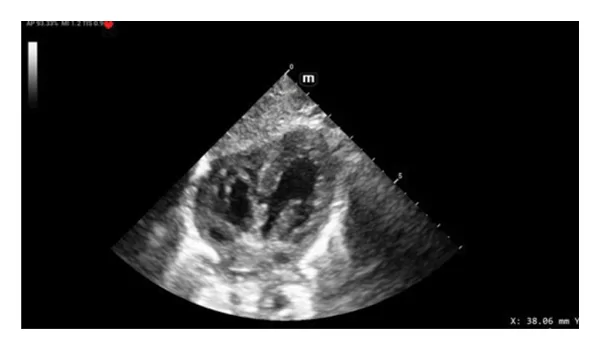
Apical four‐chamber echocardiographic view demonstrating a 4‐mm muscular ventricular septal defect (VSD). The blue arrow indicates the interventricular septal defect.

Following multidisciplinary evaluation involving pediatrics, ophthalmology, and pediatric cardiology, the patient was stabilized and managed conservatively while definitive treatment options were considered. Given the presence of multiple congenital cardiac defects and the limited availability of advanced pediatric cardiovascular services in our resource‐limited setting, the multidisciplinary team referred the patient abroad to a specialized center for further evaluation, definitive cardiac management, and long‐term follow‐up.

## 3. Discussion

Anophthalmia is a rare and severe congenital ocular malformation resulting from disruption of early ocular development during embryogenesis. Normal eye development begins during the fourth week of gestation with the formation of the optic vesicles from the forebrain. Failure at this critical stage may result in primary anophthalmia, characterized by the complete absence of the globe and optic nerve [1]. In contrast, secondary (degenerative) anophthalmia occurs when an initially formed eye subsequently regresses because of vascular insufficiency, genetic abnormalities, or intrauterine insults [8]. In the present case, neuroimaging confirmed the complete absence of both ocular globes without residual ocular tissue, supporting the diagnosis of true bilateral congenital anophthalmia [1].

Bilateral anophthalmia is substantially rarer than unilateral disease and is frequently associated with systemic congenital anomalies involving the central nervous system, craniofacial structures, endocrine system, and cardiovascular system [1, 2, 9]. Among extracranial manifestations, congenital heart defects are among the reported systemic associations, although their clinical spectrum varies considerably [1, 2]. Previous reports have described both isolated cardiac lesions and complex CHD, occurring either independently or as part of multisystem syndromic disorders. The coexistence of bilateral anophthalmia with multiple congenital cardiac defects, particularly the simultaneous presence of a VSD, ASD, and PDA, appears to be rarely reported. The occurrence of ocular and cardiac abnormalities in the same patient may suggest an underlying developmental or genetic mechanism, as both organ systems undergo critical developmental processes during embryogenesis [1, 10]. Genetic factors play an important role in the etiology of anophthalmia and microphthalmia. Pathogenic variants involving genes such as SOX2, OTX2, PAX6, RAX, and VSX2 have been implicated in abnormal ocular development and embryonic patterning [9]. Accordingly, the evaluation of bilateral anophthalmia should include consideration of an underlying genetic or chromosomal abnormality. A stepwise genetic assessment may include conventional karyotyping, chromosomal microarray analysis, and whole‐exome sequencing, depending on the clinical findings and resource availability. These investigations may identify chromosomal abnormalities or pathogenic variants associated with ocular developmental disorders and facilitate the distinction between syndromic and nonsyndromic disease [6, 11]. Establishing a molecular diagnosis may also provide important prognostic information, guide multidisciplinary management, facilitate genetic counseling, and support assessment of recurrence risk in future pregnancies. In the present case, comprehensive genetic investigations were unavailable because of resource limitations. Although the absence of a family history, parental consanguinity, and recognized antenatal risk factors may suggest a sporadic presentation, an underlying genetic etiology cannot be excluded.

Bilateral congenital anophthalmia and severe microphthalmia may occur as isolated abnormalities or as components of genetic and chromosomal syndromes. Therefore, careful evaluation for associated craniofacial, neurological, cardiac, renal, genital, skeletal, and other systemic abnormalities is essential. Important syndromic differential diagnoses include SOX2 anophthalmia syndrome, Fraser syndrome, Lenz microphthalmia syndrome, Trisomy 13, Wolf–Hirschhorn syndrome, CHARGE syndrome, Matthew–Wood syndrome, and oculoauriculofrontonasal syndrome. Recognition of characteristic phenotypic features may facilitate targeted genetic investigations, guide multidisciplinary management, and inform genetic counseling. The principal syndromic conditions relevant to the differential diagnosis of bilateral congenital anophthalmia and their distinguishing clinical features are summarized in Table 1 [10–12, 14–18]. In our patient, the absence of additional craniofacial dysmorphism, ear abnormalities, limb or digital anomalies, genital abnormalities, and other major extracardiac malformations reduced the clinical suspicion of a readily recognizable syndromic disorder. Nevertheless, an underlying syndromic etiology cannot be definitively excluded in the absence of comprehensive molecular genetic testing.

**TABLE 1:** Major syndromic differential diagnoses of bilateral congenital anophthalmia.

Syndrome	Key associated features
1. SOX2 anophthalmia syndrome [12]	Bilateral anophthalmia/microphthalmia, developmental delay, seizures, genital abnormalities, esophageal atresia.
2. Fraser syndrome [10]	Cryptophthalmos, syndactyly, genital anomalies, renal agenesis, laryngeal malformations.
3. Lenz microphthalmia syndrome [11]	Microphthalmia/anophthalmia, intellectual disability, skeletal abnormalities, dental anomalies, ear malformations.
4. Trisomy 13 [13]	Microphthalmia/anophthalmia, cleft lip and palate, polydactyly, congenital heart defects, severe developmental impairment.
5. Wolf–Hirschhorn syndrome [14]	Growth restriction, intellectual disability, seizures, characteristic facial features, ocular anomalies.
6. CHARGE syndrome [15]	Coloboma, heart defects, choanal atresia, growth retardation, genital anomalies, ear abnormalities.
7. Matthew–Wood syndrome [16]	Anophthalmia/microphthalmia, pulmonary hypoplasia, diaphragmatic defects, cardiac anomalies.
8. Oculoauriculofrontonasal syndrome [17]	Anophthalmia/microphthalmia, craniofacial asymmetry, ear anomalies

Only a limited number of reports have described bilateral congenital anophthalmia associated with CHD. Wang et al. reported two patients with anophthalmia and CHD. One patient had bilateral anophthalmia associated with complex CHD, heterotaxy, polysplenia, tracheoesophageal fistula, and multiple extracardiac anomalies, whereas the second had bilateral anophthalmia associated with an isolated large VSD [13]. Similarly, Ríos‐Méndez et al. described a neonate with bilateral anophthalmia associated with complex CHD, pulmonary hypertension, polysplenia, craniofacial dysmorphism, and additional congenital anomalies [19]. In contrast to these previously reported cases, our patient had MRI‐confirmed bilateral congenital anophthalmia associated with the simultaneous presence of a 4‐mm muscular VSD, a 4‐mm secundum ASD with an atrial septal aneurysm, and a 3‐mm PDA with restrictive left‐to‐right shunting. Collectively, these defects resulted in clinically significant left‐to‐right shunt physiology. The patient had no evidence of heterotaxy, pulmonary hypertension, tracheoesophageal fistula, or other major extracardiac malformations identified during the available clinical and imaging evaluations. To the best of our knowledge, this specific combination of bilateral congenital anophthalmia and multiple cardiac defects has been rarely reported, adding to the limited literature on the spectrum of cardiovascular abnormalities associated with congenital anophthalmia.

CHD is the most common congenital anomaly worldwide, and defects such as VSD, ASD, and PDA may coexist and contribute to clinically significant left‐to‐right shunting [20]. In our patient, tachypnea during feeding, feeding difficulties, easy fatigability, and diaphoresis were consistent with the clinical consequences of significant left‐to‐right shunt physiology [21]. Importantly, the cardiac abnormalities were not clinically evident immediately after birth and became apparent during inpatient observation. This finding underscores the importance of comprehensive systemic and cardiovascular evaluation in neonates presenting with major congenital ocular abnormalities, even when they initially appear hemodynamically stable.

Imaging played a central role in establishing the diagnosis and defining the associated abnormalities in this case. MRI confirmed true bilateral anophthalmia and excluded identifiable associated intracranial malformations, while transthoracic echocardiography delineated the cardiac anatomy, including the type and size of the VSD, ASD, and PDA, and demonstrated the direction of shunting [1, 6]. In resource‐limited settings, however, inadequate antenatal anomaly screening, incomplete perinatal documentation, and limited access to advanced diagnostic services may hinder early recognition of complex congenital abnormalities. These challenges were evident in our patient, for whom detailed antenatal imaging findings and Apgar scores were unavailable.

The management of bilateral congenital anophthalmia requires a multidisciplinary approach involving pediatrics, ophthalmology, cardiology, clinical genetics, and, when available, oculoplastic and rehabilitative services [8]. Early orbital expansion using conformers or prosthetic devices may be considered to promote orbital growth and facial symmetry, although these interventions require specialized expertise and long‐term follow‐up [10]. Similarly, the management of hemodynamically significant CHD may require medical therapy, catheter‐based intervention, or cardiac surgery, depending on the anatomy and clinical severity of the defects [22]. In settings where advanced pediatric cardiac services are unavailable, timely referral to specialized centers is essential.

The decision to refer our patient abroad for definitive cardiac management reflects the challenges encountered in many resource‐limited health systems, where advanced pediatric cardiovascular services, comprehensive genetic testing, and specialized multidisciplinary care may not be readily accessible. This case, therefore, highlights not only a rarely reported association of congenital ocular and cardiac abnormalities but also the broader challenges involved in diagnosing and managing complex congenital disorders in low‐resource settings. Strengthening antenatal screening programs, improving access to diagnostic imaging and genetic services, and establishing effective regional referral pathways may facilitate earlier diagnosis and improve continuity of care for affected patients.

This case has several limitations. First, comprehensive genetic investigations, including chromosomal microarray analysis and whole‐exome sequencing, were unavailable; therefore, an underlying genetic or syndromic etiology could not be definitively established. Second, detailed prenatal imaging records and antenatal anomaly screening results were unavailable because the patient was born in a resource‐limited rural setting, limiting assessment of the prenatal evolution of the congenital abnormalities. Third, long‐term follow‐up information after referral abroad was unavailable, precluding assessment of subsequent cardiac management, developmental progress, and long‐term outcomes. Despite these limitations, this report contributes to the limited literature describing bilateral congenital anophthalmia associated with multiple congenital cardiac defects and emphasizes the importance of comprehensive systemic evaluation and multidisciplinary management.

In conclusion, bilateral congenital anophthalmia associated with multiple congenital cardiac defects is a rarely reported clinical association. This case emphasizes the importance of comprehensive systemic evaluation, particularly cardiovascular assessment, in neonates presenting with congenital anophthalmia. Careful evaluation for associated syndromic features and consideration of genetic testing are also important for establishing the underlying etiology, guiding multidisciplinary care, and providing appropriate genetic counseling. In resource‐limited settings, early recognition and timely referral to specialized centers are particularly important when advanced diagnostic and therapeutic services are not locally available.

## 4. Conclusion

Bilateral congenital anophthalmia associated with multiple congenital cardiac defects is a rarely reported clinical association. This case highlights the importance of comprehensive neonatal screening and systemic evaluation in infants with congenital ocular malformations, particularly anophthalmia, as significant associated abnormalities may not be clinically apparent at birth. Early recognition through careful clinical assessment and appropriate imaging facilitates timely multidisciplinary management. In resource‐limited settings, limited access to antenatal screening, advanced diagnostic facilities, genetic testing, and specialized surgical services may complicate the management of such patients, emphasizing the importance of multidisciplinary collaboration and timely referral to specialized centers. Reporting such cases contributes to the limited literature on this rare association and may improve the understanding of the potential developmental and genetic mechanisms underlying congenital ocular and cardiac abnormalities.

## Author Contributions

Abdullahi Ali Roble, Abdisalam Ismail Hassan, and Ahmed Muhamad Bashir contributed to the conceptualization of the case report.

Abdisalam Ismail Hassan, Said Abdi Mohamed, and Hassan Muhumed Mohamed developed the methodology and guided the clinical approach.

Ishak Ahmed Abdi and Abdijalil Abdullahi Ali handled software‐related tasks and supported data organization.

Mohamed Osman Dahir Alasow and Shuayb Abdullahi Adan contributed to validation of the findings and case verification.

Ahmed Shafie Aden and Hanan Hassan Hirei Abdalla performed the formal analysis and interpretation of the data.

Ishak Ahmed Abdi, Abdijalil Abdullahi Ali, and Balqiz Mohamed Kulle were involved in investigation and data collection.

Balqiz Mohamed Kulle and Hassan Muhumed Mohamed managed data curation.

Abdisalam Ismail Hassan, Balqiz Mohamed Kulle, and Said Abdi Mohamed contributed to writing the original draft of the manuscript.

Abdullahi Ali Roble, Ahmed Muhamad Bashir, and Hassan Muhumed Mohamed contributed to reviewing and editing the manuscript.

Ahmed Muhamad Bashir and Abdullahi Ali Roble supervised the overall work.

Abdullahi Ali Roble managed project administration.

## Funding

The case has not received any funding.

## Disclosure

All authors have read and approved the final manuscript and agree to be accountable for all aspects of the work.

## Ethics Statement

At our institute, ethical approval is not required for the publication of case reports; therefore, our hospital is exempt from this requirement, and informed written consent was obtained from the patient to use their information and publish the case report, ensuring that identifying data remains anonymous.

## Consent

Please see the Ethics Statement.

## Conflicts of Interest

The authors declare no conflicts of interest.

## Supporting Information

Additional supporting information can be found online in the Supporting Information section.

## Supporting information


**Supporting Information** CARE‐C∼1.

## Data Availability

The article includes all relevant data to support the findings of this case report. Additional details are available from the corresponding author on reasonable request.
